# Supplementary material for: Cell-wall remodeling drives engulfment during Bacillus subtilis sporulation
Source: eLife. 2016 Nov 17;5:e18657. doi: 10.7554/eLife.18657 (PMC5158138; doi:10.7554/eLife.18657)
Supplement: Supplementary file 1. — DOI: http://dx.doi.org/10.7554/eLife.18657.025 [file elife-18657-supp1.docx]

**Plasmid construction**

**pJLG78**. This plasmid was constructed by assembling the following 4 fragments by Gibson Assembly (New England Bioloabs): (i) a fragment of 914 bp amplified with primers JLG-251 and JLG-252 from gemomic DNA of *B. subtilis* PY79, including the first 86 nucleotides of *spoIIQ* coding sequence and the 828 nucleotides immediately upstream of it; (ii) a, erythromycin resistance gene amplified with primers JLG-253 and JLG-254 from pDG1731 (Guérout-Fleury et al., 1996); (iii) a fragment of 931 bp encompassing the last 70 nucleotides of *spoIIQ* coding sequence and the region immediately downstream of it, amplified with primers JLG-249 and JLG-250 from genomic DNA of *B*. *subtilis* PY79; and (iv) a DNA fragment encompassing the spectinomycin resistant gene, the origin of replication, and the ampicillin resistant gene from pDG1662 (Guérout-Fleury et al., 1996), amplified with primers JLG-95 and JLG-96.

**pJLG88.** *pbpF* coding sequence was amplified with primers JLG-299 and JLG-300, and inserted in pJLG13 (Shin et al, 2015) amplified with JLG-139 and JLG-153 by Gibson assembly. After assembly *pbpF* is under de control of *spoIIQ* promoter.

**pJLG89.** *pbpF* coding sequence was amplified with primers JLG-300 and JLG-301, and inserted in pMDS78 (Sharp and Pogliano, 2002) amplified with JLG-139 and JLG-297 by Gibson assembly. After assembly *pbpF* is under de control of *spoIIR* promoter.

**pJLG90.** *pbpF* coding sequence was amplified with primers JLG-302 and JLG-303, and inserted in pJLG20 (Shin et al, 2015) amplified with JLG-138 and JLG-152 by Gibson assembly. After assembly *pbpF* is under de control of *spoIID* promoter.

**pJLG91.** *pbpG* coding sequence was amplified with primers JLG-304 and JLG-305, and inserted in pJLG13 (Shin et al, 2015) amplified with JLG-139 and JLG-153 by Gibson assembly. After assembly *pbpG* is under de control of *spoIIQ* promoter.

**pJLG92.** *pbpG* coding sequence was amplified with primers JLG-305 and JLG-306, and inserted in pMDS78 (Sharp and Pogliano, 2002) amplified with JLG-139 and JLG-297 by Gibson assembly. After assembly *pbpG* is under de control of *spoIIR* promoter.

**pJLG93.** *pbpG* coding sequence was amplified with primers JLG-307 and JLG-308, and inserted in pJLG20 (Shin et al, 2015) amplified with JLG-138 and JLG-152 by Gibson assembly. After assembly *pbpG* is under de control of *spoIID* promoter.

**pJLG213.** pJLG88 was amplified with primers JLG-153 and JLG-889, and assembled with *sfGFP* amplified with primers JLG-891 and JLG-894 from pJLG36 (Shin et al, 2015) by Gibson assembly (New England Biolabs). After assembly *sfGFP* coding sequence is fused to the N-terminus of *pbpF* coding sequence, and both coding sequences are separated by a linker consisting of eight alternating alanines and serines. The fusion gene is under de control of *spoIIQ* promoter.

**pJLG214.** pJLG89 was amplified with primers JLG-297 and JLG-889, and assembled with *sfGFP* amplified with primers JLG-892 and JLG-894 from pJLG36 (Shin et al, 2015) by Gibson assembly (New England Biolabs). After assembly *sfGFP* coding sequence fused to the N-terminus of *pbpF* coding sequence, and both sequences are separated by a linker consisting of eight alternating alanines and serines. The fusion gene is under de control of *spoIIR* promoter.

**pJLG215.** pJLG90 was amplified with primers JLG-152 and JLG-889, and assembled with *sfGFP* amplified with primers JLG-893 and JLG-894 from pJLG-36 (Shin et al., 2015) by Gibson assembly (New England Biolabs). After assembly *sfGFP* coding sequence is fused to the N-terminus of *pbpF* coding sequence, and both sequences are separated by a linker consisting of eight alternating alanines and serines. The fusion gene is under de control of *spoIID* promoter.

**pJLG218.** pJLG91 was amplified with primers JLG-153 and JLG-890, and assembled with *sfGFP* amplified with primers JLG-891 and JLG-894 from pJLG36 (Shin et al, 2015) by Gibson assembly (New England Biolabs). After assembly *sfGFP* coding sequence is in fused to the N-terminus of *pbpG* coding sequence, and both sequences are separated by a linker consisting of eight alternating alanines and serines. The fusion gene is under de control of *spoIIQ* promoter.

**pJLG219.** pJLG92 was amplified with primers JLG-297 and JLG-890, and assembled with *sfGFP* amplified with primers JLG-892 and JLG-894 from pJLG36 (Shin et al, 2015) by Gibson assembly (New England Biolabs). After assembly *sfGFP* coding sequence is fused to the N-teminus of *pbpG* coding sequence, and both sequences are separated by a linker consisting of eight alternating alanines and serines. The fusion gene is under de control of *spoIIR* promoter.

**pJLG220.** pJLG93 was amplified with primers JLG-152 and JLG-890, and assembled with *sfGFP* amplified with primers JLG-893 and JLG-894 from pJLG36 (Shin et al, 2015) by Gibson assembly (New England Biolabs). After assembly *sfGFP* coding sequence is fused to the N-terminus of *pbpG* coding sequence, and both sequences are separated by a linker consisting of eight alternating alanines and serines. The fusion gene is under de control of *spoIID* promoter.

**pJLG222.** *ponA* coding sequence was amplified with primers JLG-977 and JLG-978, and inserted in pJLG213 amplified with JLG-139 and JLG-453 by Gibson assembly. After assembly *sfGFP* coding sequence is fused to the N-terminus of *ponA* coding sequence, and both sequences are separated by a linker consisting of eight alternating alanines and serines. The fusion gene is under de control of *spoIIQ* promoter.

**pJLG223.** *ponA* coding sequence was amplified with primers JLG-977 and JLG-978, and inserted in pJLG214 amplified with JLG-139 and JLG-453 by Gibson assembly. After assembly *sfGFP* coding sequence is fused to the N-terminus of *ponA* coding sequence, and both sequences are separated by a linker consisting of eight alternating alanines and serines. The fusion gene is under de control of *spoIIR* promoter.

**pJLG224.** *pbpD* coding sequence was amplified with primers JLG-980 and JLG-981, and inserted in pJLG213 amplified with JLG-139 and JLG-453 by Gibson assembly. After assembly *sfGFP* coding sequence is fused to the N-terminus of *pbpD* coding sequence, and both sequences are separated by a linker consisting of eight alternating alanines and serines. The fusion gene is under de control of *spoIIQ* promoter.

**pJLG225.** *pbpD* coding sequence was amplified with primers JLG-980 and JLG-981, and inserted in pJLG214 amplified with JLG-139 and JLG-453 by Gibson assembly. After assembly *sfGFP* coding sequence is fused to the N-terminus of *pbpD* coding sequence, and both sequences are separated by a linker consisting of eight alternating alanines and serines. The fusion gene is under de control of *spoIIR* promoter.

**pJLG226.** *pbpD* coding sequence was amplified with primers JLG-980 and JLG-982, and inserted in pJLG220 amplified with JLG-138 and JLG-453 by Gibson assembly. After assembly *sfGFP* coding sequence is fused to the N-terminus of *pbpD* coding sequence, and both sequences are separated by a linker consisting of eight alternating alanines and serines. The fusion gene is under de control of *spoIID* promoter.

**pJLG230.** *ponA* coding sequence was amplified with primers JLG-977 and JLG-979, and inserted in pJLG220 amplified with JLG-138 and JLG-453 by Gibson assembly. After assembly *sfGFP* coding sequence is fused to the N-terminus of *ponA* coding sequence, and both sequences are separated by a linker consisting of eight alternating alanines and serines. The fusion gene is under de control of *spoIID* promoter.

**pJLG263.** *pbpB* coding sequence was amplified with primers JLG-1021 and JLG-1022, and inserted in pJLG213 amplified with JLG-139 and JLG-453 by Gibson assembly. After assembly *sfGFP* coding sequence is fused to the N-terminus of *pbpB* coding sequence, and both sequences are separated by a linker consisting of eight alternating alanines and serines. The fusion gene is under de control of *spoIIQ* promoter.

**pJLG264.** *pbpB* coding sequence was amplified with primers JLG-1021 and JLG-1022, and inserted in pJLG214 amplified with JLG-139 and JLG-453 by Gibson assembly. After assembly *sfGFP* coding sequence is fused to the N-terminus of *pbpB* coding sequence, and both sequences are separated by a linker consisting of eight alternating alanines and serines. The fusion gene is under de control of *spoIIR* promoter.

**pJLG265.** *pbpB* coding sequence was amplified with primers JLG-1021 and JLG-1023, and inserted in pJLG220 amplified with JLG-138 and JLG-453 by Gibson assembly. After assembly *sfGFP* coding sequence is fused to the N-terminus of *pbpB* coding sequence, and both sequences are separated by a linker consisting of eight alternating alanines and serines. The fusion gene is under de control of *spoIID* promoter.

**pJLG266.** *pbpH* coding sequence was amplified with primers JLG-1027 and JLG-1028, and inserted in pJLG213 amplified with JLG-139 and JLG-453 by Gibson assembly. After assembly *sfGFP* coding sequence is fused to the N-terminus of *pbpH* coding sequence, and both sequences are separated by a linker consisting of eight alternating alanines and serines. The fusion gene is under de control of *spoIIQ* promoter.

**pJLG267.** *pbpH* coding sequence was amplified with primers JLG-1027 and JLG-1028, and inserted in pJLG214 amplified with JLG-139 and JLG-453 by Gibson assembly. After assembly *sfGFP* coding sequence is fused to the N-terminus of *pbpH* coding sequence, and both sequences are separated by a linker consisting of eight alternating alanines and serines. The fusion gene is under de control of *spoIIR* promoter.

**pJLG268.** *pbpH* coding sequence was amplified with primers JLG-1027 and JLG-1029, and inserted in pJLG220 amplified with JLG-138 and JLG-453 by Gibson assembly. After assembly *sfGFP* coding sequence is fused to the N-terminus of *pbpH* coding sequence, and both sequences are separated by a linker consisting of eight alternating alanines and serines. The fusion gene is under de control of *spoIID* promoter.

**pJLG269.** *pbpI* coding sequence was amplified with primers JLG-1030 and JLG-1031, and inserted in pJLG213 amplified with JLG-139 and JLG-453 by Gibson assembly. After assembly *sfGFP* coding sequence is fused to the N-terminus of *pbpI* coding sequence, and both sequences are separated by a linker consisting of eight alternating alanines and serines. The fusion gene is under de control of *spoIIQ* promoter.

**pJLG270.** *pbpI* coding sequence was amplified with primers JLG-1030 and JLG-1031, and inserted in pJLG214 amplified with JLG-139 and JLG-453 by Gibson assembly. After assembly *sfGFP* coding sequence is fused to the N-terminus of *pbpI* coding sequence, and both sequences are separated by a linker consisting of eight alternating alanines and serines. The fusion gene is under de control of *spoIIR* promoter.

**pJLG271.** *pbpI* coding sequence was amplified with primers JLG-1030 and JLG-1032, and inserted in pJLG220 amplified with JLG-138 and JLG-453 by Gibson assembly. After assembly *sfGFP* coding sequence is fused to the N-terminus of *pbpI* coding sequence, and both sequences are separated by a linker consisting of eight alternating alanines and serines. The fusion gene is under de control of *spoIID* promoter.

**pJLG272.** *pbpX* coding sequence was amplified with primers JLG-1033 and JLG-1034, and inserted in pJLG213 amplified with JLG-139 and JLG-453 by Gibson assembly. After assembly *sfGFP* coding sequence is fused to the N-terminus of *pbpX* coding sequence, and both sequences are separated by a linker consisting of eight alternating alanines and serines. The fusion gene is under de control of *spoIIQ* promoter.

**pJLG273.** *pbpX* coding sequence was amplified with primers JLG-1033 and JLG-1034, and inserted in pJLG214 amplified with JLG-139 and JLG-453 by Gibson assembly. After assembly *sfGFP* coding sequence is fused to the N-terminus of *pbpX* coding sequence, and both sequences are separated by a linker consisting of eight alternating alanines and serines. The fusion gene is under de control of *spoIIR* promoter.

**pJLG274.** *pbpX* coding sequence was amplified with primers JLG-1033 and JLG-1035, and inserted in pJLG220 amplified with JLG-138 and JLG-453 by Gibson assembly. After assembly *sfGFP* coding sequence is fused to the N-terminus of *pbpX* coding sequence, and both sequences are separated by a linker consisting of eight alternating alanines and serines. The fusion gene is under de control of *spoIID* promoter.

**pJLG275.** *pbpA* coding sequence was amplified with primers JLG-1036 and JLG-1037, and inserted in pJLG213 amplified with JLG-139 and JLG-453 by Gibson assembly. After assembly *sfGFP* coding sequence is fused to the N-terminus of *pbpA* coding sequence, and both sequences are separated by a linker consisting of eight alternating alanines and serines. The fusion gene is under de control of *spoIIQ* promoter.

**pJLG276.** *pbpA* coding sequence was amplified with primers JLG-1036 and JLG-1037, and inserted in pJLG214 amplified with JLG-139 and JLG-453 by Gibson assembly. After assembly *sfGFP* coding sequence is fused to the N-terminus of *pbpA* coding sequence, and both sequences are separated by a linker consisting of eight alternating alanines and serines. The fusion gene is under de control of *spoIIR* promoter.

**pJLG277.** *pbpA* coding sequence was amplified with primers JLG-1036 and JLG-1038, and inserted in pJLG220 amplified with JLG-138 and JLG-453 by Gibson assembly. After assembly *sfGFP* coding sequence is fused to the N-terminus of *pbpA* coding sequence, and both sequences are separated by a linker consisting of eight alternating alanines and serines. The fusion gene is under de control of *spoIID* promoter.

**pJLG278.** *dacA* coding sequence was amplified with primers JLG-1039 and JLG-1040, and inserted in pJLG213 amplified with JLG-139 and JLG-453 by Gibson assembly. After assembly *sfGFP* coding sequence is fused to the N-terminus of *dacA* coding sequence, and both sequences are separated by a linker consisting of eight alternating alanines and serines. The fusion gene is under de control of *spoIIQ* promoter.

**pJLG279.** *dacA* coding sequence was amplified with primers JLG-1039 and JLG-1040, and inserted in pJLG214 amplified with JLG-139 and JLG-453 by Gibson assembly. After assembly *sfGFP* coding sequence is fused to the N-terminus of *dacA* coding sequence, and both sequences are separated by a linker consisting of eight alternating alanines and serines. The fusion gene is under de control of *spoIIR* promoter.

**pJLG280.** *dacA* coding sequence was amplified with primers JLG-1039 and JLG-1041, and inserted in pJLG220 amplified with JLG-138 and JLG-453 by Gibson assembly. After assembly *sfGFP* coding sequence is fused to the N-terminus of *dacA* coding sequence, and both sequences are separated by a linker consisting of eight alternating alanines and serines. The fusion gene is under de control of *spoIID* promoter.

**pJLG281.** *dacB* coding sequence was amplified with primers JLG-1042 and JLG-1043, and inserted in pJLG213 amplified with JLG-139 and JLG-453 by Gibson assembly. After assembly *sfGFP* coding sequence is fused to the N-terminus of *dacB* coding sequence, and both sequences are separated by a linker consisting of eight alternating alanines and serines. The fusion gene is under de control of *spoIIQ* promoter.

**pJLG282.** *dacB* coding sequence was amplified with primers JLG-1042 and JLG-1043, and inserted in pJLG214 amplified with JLG-139 and JLG-453 by Gibson assembly. After assembly *sfGFP* coding sequence is fused to the N-terminus of *dacB* coding sequence, and both sequences are separated by a linker consisting of eight alternating alanines and serines. The fusion gene is under de control of *spoIIR* promoter.

**pJLG283.** *dacB* coding sequence was amplified with primers JLG-1042 and JLG-1044, and inserted in pJLG220 amplified with JLG-138 and JLG-453 by Gibson assembly. After assembly *sfGFP* coding sequence is fused to the N-terminus of *dacB* coding sequence, and both sequences are separated by a linker consisting of eight alternating alanines and serines. The fusion gene is under de control of *spoIID* promoter.

**pJLG284.** *dacC* coding sequence was amplified with primers JLG-1045 and JLG-1046, and inserted in pJLG213 amplified with JLG-139 and JLG-453 by Gibson assembly. After assembly *sfGFP* coding sequence is fused to the N-terminus of *dacC* coding sequence, and both sequences are separated by a linker consisting of eight alternating alanines and serines. The fusion gene is under de control of *spoIIQ* promoter.

**pJLG285.** *dacC* coding sequence was amplified with primers JLG-1045 and JLG-1046, and inserted in pJLG214 amplified with JLG-139 and JLG-453 by Gibson assembly. After assembly *sfGFP* coding sequence is fused to the N-terminus of *dacC* coding sequence, and both sequences are separated by a linker consisting of eight alternating alanines and serines. The fusion gene is under de control of *spoIIR* promoter.

**pJLG286.** *dacC* coding sequence was amplified with primers JLG-1045 and JLG-1047, and inserted in pJLG220 amplified with JLG-138 and JLG-453 by Gibson assembly. After assembly *sfGFP* coding sequence is fused to the N-terminus of *dacC* coding sequence, and both sequences are separated by a linker consisting of eight alternating alanines and serines. The fusion gene is under de control of *spoIID* promoter.

**pJLG287.** *dacF* coding sequence was amplified with primers JLG-1048 and JLG-1049, and inserted in pJLG213 amplified with JLG-139 and JLG-453 by Gibson assembly. After assembly *sfGFP* coding sequence is fused to the N-terminus of *dacF* coding sequence, and both sequences are separated by a linker consisting of eight alternating alanines and serines. The fusion gene is under de control of *spoIIQ* promoter.

**pJLG288.** *dacF* coding sequence was amplified with primers JLG-1048 and JLG-1049, and inserted in pJLG214 amplified with JLG-139 and JLG-453 by Gibson assembly. After assembly *sfGFP* coding sequence is fused to the N-terminus of *dacF* coding sequence, and both sequences are separated by a linker consisting of eight alternating alanines and serines. The fusion gene is under de control of *spoIIR* promoter.

**pJLG289.** *dacF* coding sequence was amplified with primers JLG-1048 and JLG-1050, and inserted in pJLG220 amplified with JLG-138 and JLG-453 by Gibson assembly. After assembly *sfGFP* coding sequence is fused to the N-terminus of *dacF* coding sequence, and both sequences are separated by a linker consisting of eight alternating alanines and serines. The fusion gene is under de control of *spoIID* promoter.

**pJLG296.** *pbpE* coding sequence was amplified with primers JLG-1024 and JLG-1025, and inserted in pJLG213 amplified with JLG-139 and JLG-453 by Gibson assembly. After assembly *sfGFP* coding sequence is fused to the N-terminus of *pbpE* coding sequence, and both sequences are separated by a linker consisting of eight alternating alanines and serines. The fusion gene is under de control of *spoIIQ* promoter.

**pJLG298.** *pbpE* coding sequence was amplified with primers JLG-1024 and JLG-1025, and inserted in pJLG214 amplified with JLG-139 and JLG-453 by Gibson assembly. After assembly *sfGFP* coding sequence is fused to the N-terminus of *pbpE* coding sequence, and both sequences are separated by a linker consisting of eight alternating alanines and serines. The fusion gene is under de control of *spoIIR* promoter.

**pJLG299.** *pbpE* coding sequence was amplified with primers JLG-1024 and JLG-1026, and inserted in pJLG220 amplified with JLG-138 and JLG-453 by Gibson assembly. After assembly *sfGFP* coding sequence is fused to the N-terminus of *pbpE* coding sequence, and both sequences are separated by a linker consisting of eight alternating alanines and serines. The fusion gene is under de control of *spoIID* promoter.

**pJLG361**. This plasmid was constructed by assembling the following 4 fragments by Gibson Assembly (New England Bioloabs): (i) a fragment of 662 bp amplified with primers JLG-1312 and JLG-1313 from gemomic DNA of *B. subtilis* PY79, including the first 50 nucleotides of *gerM* coding sequence and the 512 nucleotides immediately upstream of it; (ii) a kanamycin resistance gene amplified with primers oER421 and oER422 from pJLG3 (Shin et al, 2015); (iii) a fragment of 648 bp encompassing the last 38 nucleotides of *gerM* coding sequence and the region immediately downstream of it, amplified with primers JLG-1314 and JLG-1315 from genomic DNA of *B*. *subtilis* PY79; and (iv) a DNA fragment encompassing the spectinomycin resistant gene, the origin of replication, and the ampicillin resistant gene from pDG1662 (Guérout-Fleury et al., 1996), amplified with primers JLG-95 and JLG-96.

**pJLG363.** *mreB* coding sequence was amplified with primers JLG-1330 and JLG-1331, and inserted in pJLG213 amplified with JLG-139 and JLG-453 by Gibson assembly. After assembly *sfGFP* coding sequence is fused to the N-terminus of *mreB* coding sequence, and both sequences are separated by a linker consisting of eight alternating alanines and serines. The fusion gene is under de control of *spoIIQ* promoter.

**pJLG364.** *mreB* coding sequence was amplified with primers JLG-1330 and JLG-1331, and inserted in pJLG214 amplified with JLG-139 and JLG-453 by Gibson assembly. After assembly *sfGFP* coding sequence is fused to the N-terminus of *mreB* coding sequence, and both sequences are separated by a linker consisting of eight alternating alanines and serines. The fusion gene is under de control of *spoIIR* promoter.

**pJLG365.** *mreB* coding sequence was amplified with primers JLG-1330 and JLG-1332, and inserted in pJLG220 amplified with JLG-138 and JLG-453 by Gibson assembly. After assembly *sfGFP* coding sequence is fused to the N-terminus of *mreB* coding sequence, and both sequences are separated by a linker consisting of eight alternating alanines and serines. The fusion gene is under de control of *spoIID* promoter.

**pJLG366.** *mbl* coding sequence was amplified with primers JLG-1333 and JLG-1334, and inserted in pJLG214 amplified with JLG-139 and JLG-453 by Gibson assembly. After assembly *sfGFP* coding sequence is fused to the N-terminus of *mbl* coding sequence, and both sequences are separated by a linker consisting of eight alternating alanines and serines. The fusion gene is under de control of *spoIIR* promoter.

**pJLG367.** *mbl* coding sequence was amplified with primers JLG-1333 and JLG-1335, and inserted in pJLG220 amplified with JLG-138 and JLG-453 by Gibson assembly. After assembly *sfGFP* coding sequence is fused to the N-terminus of *mbl* coding sequence, and both sequences are separated by a linker consisting of eight alternating alanines and serines. The fusion gene is under de control of *spoIID* promoter.

**pJLG368.** *mreBH* coding sequence was amplified with primers JLG-1336 and JLG-1337, and inserted in pJLG213 amplified with JLG-139 and JLG-453 by Gibson assembly. After assembly *sfGFP* coding sequence is fused to the N-terminus of *mreBH* coding sequence, and both sequences are separated by a linker consisting of eight alternating alanines and serines. The fusion gene is under de control of *spoIIQ* promoter.

**pJLG369.** *mreBH* coding sequence was amplified with primers JLG-1336 and JLG-1337, and inserted in pJLG214 amplified with JLG-139 and JLG-453 by Gibson assembly. After assembly *sfGFP* coding sequence is fused to the N-terminus of *mreBH* coding sequence, and both sequences are separated by a linker consisting of eight alternating alanines and serines. The fusion gene is under de control of *spoIIR* promoter.

**pJLG370.** *mreBH* coding sequence was amplified with primers JLG-1336 and JLG-1338, and inserted in pJLG220 amplified with JLG-138 and JLG-453 by Gibson assembly. After assembly *sfGFP* coding sequence is fused to the N-terminus of *mreBH* coding sequence, and both sequences are separated by a linker consisting of eight alternating alanines and serines. The fusion gene is under de control of *spoIID* promoter.

**pJLG371.** *mbl* coding sequence was amplified with primers JLG-1333 and JLG-1334, and inserted in pJLG213 amplified with JLG-139 and JLG-453 by Gibson assembly. After assembly *sfGFP* coding sequence is fused to the N-terminus of *mbl* coding sequence, and both sequences are separated by a linker consisting of eight alternating alanines and serines. The fusion gene is under de control of *spoIIQ* promoter.
