# Supplementary figures and images for: Cell-wall remodeling drives engulfment during Bacillus subtilis sporulation
Source: eLife. 2016 Nov 17;5:e18657. doi: 10.7554/eLife.18657 (PMC5158138; doi:10.7554/eLife.18657)

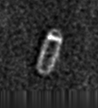

Supplement: Supplementary file 2. — DOI: http://dx.doi.org/10.7554/eLife.18657.026 [file elife-18657-supp2.zip › image_analysis_example/WT_FM464.tif]
